# Pharmacokinetic/pharmacodynamic evaluation of multidose oritavancin for prolonged durations of therapy

**DOI:** 10.1128/aac.01049-26

**Published:** 2026-10-14

**Authors:** Cecilia F. Volk, Max Blumenthal, Nolan Thomas, Wesley D. Kufel, Warren E. Rose, Sin Yin Lim

**Affiliations:** 1 University of Wisconsin, Madison – School of Pharmacy 15533, Madison, Wisconsin, USA; 2 State University of New York, Binghamton School of Pharmacy and Pharmaceutical Sciences 465824, Binghamton, New York, USA; 3 State University of New York, Upstate Medical University 12302, Syracuse, New York, USA; University of Houston, Houston, Texas, USA

**Keywords:** pharmacokinetics, pharmacodynamics, oritavancin

## Abstract

Managing multidrug-resistant gram-positive infections is challenging, especially when prolonged therapy is needed for conditions like bacteremia, endocarditis, osteomyelitis, or other deep-seated infections. Oritavancin, a long-acting lipoglycopeptide, is increasingly used in these scenarios and offers an alternative to central catheter placement. However, despite its growing use for extended treatment, optimal dosing strategies and pharmacokinetic targets remain unclear. An *in silico* pharmacokinetic/pharmacodynamic simulation was performed to assess the predicted oritavancin concentration resulting from weekly and every 2-week dosing regimens. The simulations utilized a previously published model, which utilized pharmacokinetic sampling data from two single-dose, phase 3 clinical trials. A 100% free time above the MIC was utilized as the pharmacodynamic target. The Clinical and Laboratory Standards Institute breakpoint (0.12 µg/mL) and MIC90 (0.06 µg/mL) values for both *Staphylococcus aureus* and *Enterococcus faecalis* were utilized as benchmarks. Eighty-five percent protein binding was assumed. A six-dose regimen of 1,200 mg weekly is expected to maintain free plasma concentrations above the breakpoint for a median of 10.4 weeks (lower 5th percentile 9.6) and above the MIC90 for about 1.5 weeks longer, exceeding what is usually required for acute treatment of most infections. Alternatively, using a three-dose regimen of 1,200 mg every 2 weeks is expected to maintain free plasma concentrations above the breakpoint for a median of 8.4 weeks (lower 5th percentile 7.9), with trough values remaining therapeutic between doses. Every 2-week dosing strategies are expected to maintain therapeutic concentrations between doses. This strategy may be employed to reduce the cost of treatment and improve patient convenience.

## INTRODUCTION

Invasive gram-positive infections caused by multidrug-resistant organisms such as methicillin-resistant *Staphylococcus aureus* (MRSA) or vancomycin-resistant *Enterococcus* spp. (VRE) frequently necessitate prolonged courses of antimicrobial therapy (1). Patients with deep-seated infections such as endocarditis, bacteremia, and osteomyelitis often require weeks of intravenous treatment, which can pose substantial challenges beyond antimicrobial selection alone. Maintaining vascular access, arranging outpatient infusions, and navigating cost barriers can complicate care delivery and limit feasible treatment options, particularly for patients unable to remain hospitalized for extended periods (2). Long-acting lipoglycopeptide antibiotics have gained increasing attention as potential options to mitigate these logistical barriers by allowing clinicians to deliver parenteral therapy while minimizing the need for indwelling central catheters or frequent infusion visits. This paradigm has been especially attractive for patients with complex social circumstances and has been commonly utilized in patients who inject drugs (3).

Oritavancin (ORI) is a semisynthetic lipoglycopeptide with potent activity against a range of gram-positive pathogens, including MRSA and VRE (4). It is approved as a single 1,200 mg intravenous dose for the treatment of acute bacterial skin and skin structure infections. Clinical trials supporting this indication demonstrated similar efficacy to standard multi-day comparator regimens, establishing the feasibility of single-dose therapy for these infections (5, 6). The pharmacokinetic (PK) characteristics of oritavancin include extensive tissue distribution and a prolonged terminal half-life of approximately 10–16 days (7).

These antimicrobial and pharmacologic properties have prompted increasing off-label use of ORI in infections traditionally requiring prolonged intravenous treatment courses. Observational studies and case series have described its use in a variety of serious infections, including bone and joint infections, bloodstream infections, and infective endocarditis (3, 8–14). In these settings, clinicians have employed repeated dosing strategies at variable intervals, most commonly administering additional 1,200 or 800 mg doses weekly. However, this practice has emerged largely in the absence of data to inform optimal dose selection, dosing frequency, or expected duration of effective antimicrobial exposure. To date, limited pharmacokinetic/pharmacodynamic (PK/PD) data are available to support these dosing regimens.

To address these gaps, we performed an *in silico* PK simulation using published population PK parameters to evaluate multidose ORI regimens reported in the literature, as well as alternative regimens designed to extend dosing intervals. Predicted plasma concentrations were analyzed to estimate the duration of effective therapy achieved by each regimen. These analyses aim to guide ORI dosing in prolonged treatment uses and to inform future clinical investigation.

## MATERIALS AND METHODS

An *in silico* simulation was performed to assess the predicted ORI concentrations resulting from a variety of dosing regimens. The population PK model was previously developed by Rubino et al., which utilized PK sampling data from two phase 3 clinical trials (5–7). These study patients had an average age of 45.9 years (coefficient of variation [CV]: 29.4%) and an average weight of 79.9 kg (CV: 28.8%). The resulting model had three-compartment distribution and first-order elimination with an average steady-state volume of distribution of 97.8 L and a clearance of 0.445 L/h. After infusion, ORI initially exhibits rapid and extensive tissue distribution, followed by a slow redistribution from tissue back into plasma, contributing to its long terminal half-life of approximately 10–16 days. Of note, our model utilized a 1-h infusion time, in contrast to the 3-h infusion utilized in the Rubino et al. (7) model. This infusion time is only approved for use with Kimyrsa (available as a 1,200 mg vial) and not Orbactiv (supplied in 400 mg vials) due to the addition of a solubilizing agent that allows for a lower infusion volume and shorter infusion time (4, 15). Previous PK analysis has demonstrated that the shorter infusion time leads to a higher *C*_max_ but no change in *C*_min_ or area under the curve (AUC) over the first 72 h (AUC_0–72h_) (16). These data indicate that these infusion times can likely be considered interchangeable from a PK perspective. Age and height were significant covariates for central volume of distribution and clearance, respectively. Full details of covariate relationships are available in the original publication (7).

This study first developed a PK model data set of 1,000 hypothetical patients. In the construction of the 1,000 simulated patients, 100 patients were first generated with ages and heights randomly sampled, with replacement, from truncated normal distributions based on the demographic distributions (mean, coefficient of variation [CV], and range) reported in Rubino et al. (7). The simulated patients had an average age of 47 years (CV: 25%, range: 19–75 years) and an average height of 169 cm (CV: 6.3%, range: 147–195 cm). Subsequently, each simulated patient was assigned 10 sets of PK parameters, each incorporating interindividual variability, based on the final population PK model developed by Rubino et al. Plasma concentration-time profile of each patient was simulated for four dosing regimens: (i) 1,200 mg weekly, (ii) 1,200 mg loading dose followed by 800 mg weekly, (iii) 1,200 mg every 2 weeks, and (iv) 1,200 mg loading dose followed by 800 mg every 2 weeks. Infusions were simulated to be administered over 1 h. Free plasma concentrations were calculated using an unbound fraction of 0.15 (4). NONMEM/PDx-Pop (v5.4, ICON, Dublin, Ireland) was used for the simulations. The complete NONMEM control file can be found in the Supplemental material.

Duration of target attainment was assessed for each modeled regimen using a free time above the minimum inhibitory concentration (*f*T > MIC) approach. Two MIC benchmarks were used: (i) the ORI breakpoint for *S. aureus* and vancomycin-susceptible *E. faecalis* of 0.12 µg/mL established by the Clinical and Laboratory Standards Institute (CLSI) and (ii) the 90th percentile MIC (MIC_90_) (0.06 µg/mL) for *S. aureus* and *Enterococcus* spp. reported from worldwide surveillance studies for ORI (17). Because 6 weeks is a commonly utilized duration of therapy, we next aimed to determine how many ORI doses are required to attain this target duration. For each of the previously modeled dosing strategies, simulations of fewer total doses were developed. Simulated regimens were deemed adequate to achieve this duration if the 5th percentile free plasma concentration at 6 weeks was above the breakpoint (0.12 µg/mL).

## RESULTS

**Fig 1:**
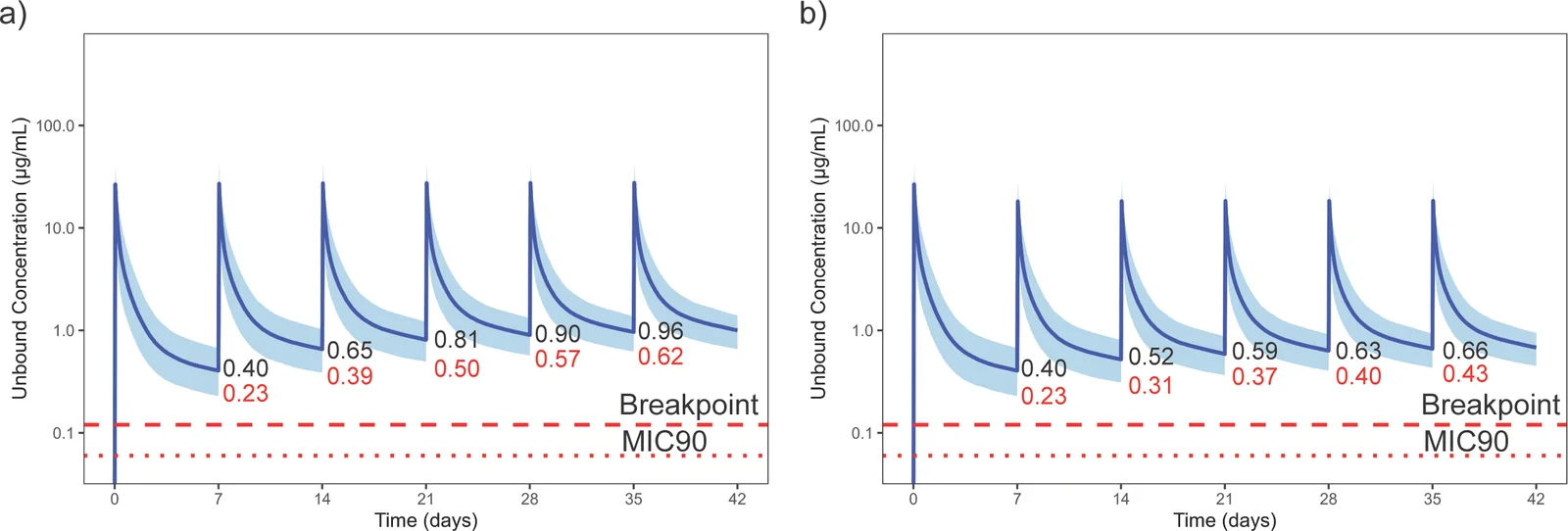
Simulated oritavancin (ORI) exposure resulting from weekly dosing. Free plasma concentrations after administration of ORI 1,200 mg weekly for six doses (**a**) or ORI 1,200 mg on day 0 and then 800 mg weekly for five doses (**b**). Solid lines indicate the median value and shaded region highlights the 5th–95th percentiles. The breakpoint (0.12 µg/mL) and 90th percentile MIC (MIC_90_) (0.06 µg/mL) are indicated by red dashed lines. Median (black) and lower 5th percentile (red) trough values (µg/mL) are listed for each dose.

The first two simulated dosing regimens were 1,200 mg weekly and a 1,200 mg loading dose followed by 800 mg weekly. Both were modeled as a six-dose regimen, aimed to simulate what clinicians may intend to be a 6-week treatment duration. These weekly regimens are the two most reported in published case series (3, 8–14). The predicted free plasma concentrations resulting from these regimens are shown in Fig. 1, with predicted *f*T > MIC listed in Table 1. Six doses of 1,200 mg administered weekly resulted in predicted free plasma concentrations above the breakpoint for a median of 10.4 weeks (5th percentile 9.6 weeks) and above the MIC_90_ for a median of 11.9 weeks (5th percentile 10.7 weeks), far exceeding the intended 6-week duration of therapy. Reduced dosing of 1,200 mg followed by 800 mg weekly resulted in predicted free plasma concentrations above the breakpoint for a median of 9.6 weeks (5th percentile 9.0 weeks) and above the MIC_90_ for a median of 11.0 weeks (5th percentile 10.1 weeks), a reduction in duration of therapy of less than a week.

**TABLE 1:** Time of target achievement for each simulated regimen^*a*^

Regimen	Weeks with free serum concentration > MIC (median [5th to 95th percentile])	
	MIC_90_ (0.06 µg/mL)	Breakpoint (0.12 µg/mL)
1,200 mg weekly × 6 doses	11.9 (10.7–13.1)	10.4 (9.6–11.3)
1,200 mg day 0, 800 mg weekly × 5 doses	11.0 (10.1–12.2)	9.6 (9.0–10.3)
1,200 mg every 2 weeks × 3 doses	9.8 (9.0–10.8)	8.4 (7.9–9.0)
1,200 mg day 0, 800 mg every 2 weeks × 2 doses	9.1 (8.4–9.8)	7.6 (7.1–8.1)

^*a*^ MIC, minimum inhibitory concentration; MIC_90_; 90th percentile MIC.

**Fig 2:**
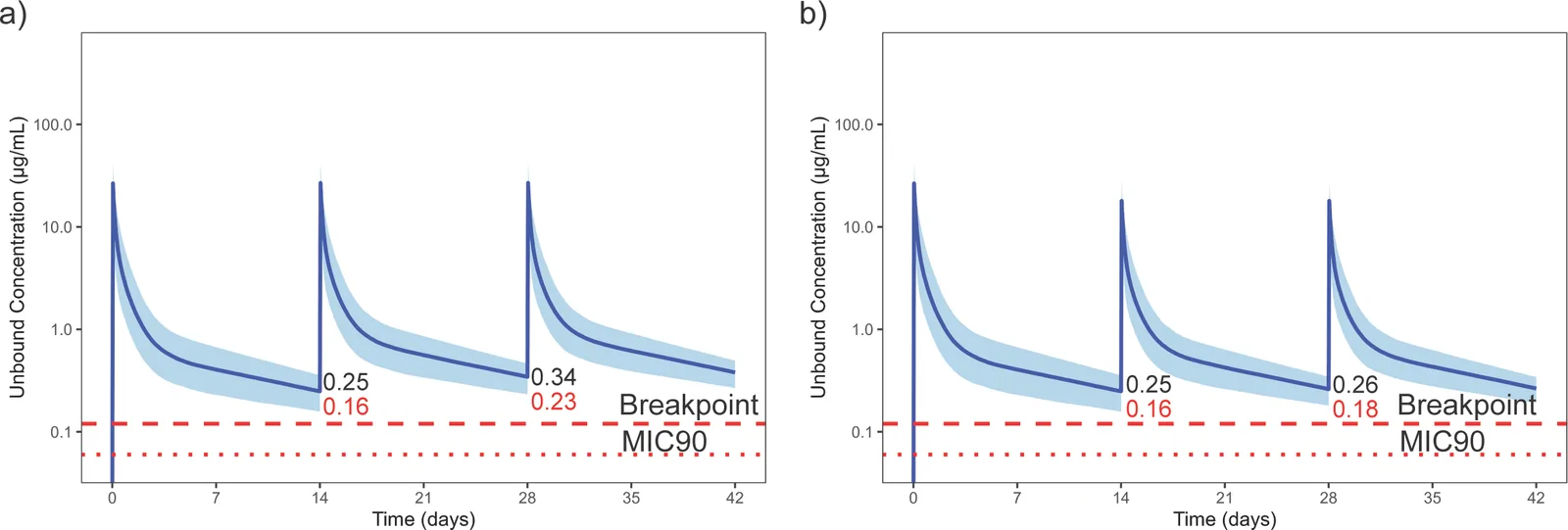
Simulated oritavancin (ORI) exposure resulting from every 2-week dosing. Free plasma concentrations after administration of ORI 1,200 mg every 2 weeks for three doses (**a**) or ORI 1,200 mg on day 0 and then 800 mg every 2 weeks for two doses (**b**). Solid lines indicate the median value, and the shaded region highlights the 5th–95th percentiles. The breakpoint (0.12 µg/mL) and 90th percentile MIC (MIC90) (0.06 µg/mL) are indicated by red dashed lines. Median (black) and lower 5th percentile (red) trough values (µg/mL) are listed for each dose.

The elevated plasma concentrations between doses and extended total duration of therapy (beyond 6 weeks) resulting from these regimens suggest the possibility of using an every 2-week dosing scheme without compromising target attainment. Using this approach, the next two simulated regimens were 1,200 mg every 14 days or a 1,200 mg loading dose followed by 800 mg every 14 days, both administered for a total of three doses. These regimens have the potential to reduce both drug and administration costs, while improving patient convenience. The predicted free plasma concentrations resulting from these regimens are shown in Fig. 2, with predicted *f*T > MIC listed in Table 1. Three doses of 1,200 mg administered every 2 weeks resulted in predicted free plasma concentrations above the breakpoint for a median of 8.4 weeks (5th percentile 7.9 weeks) and above the MIC_90_ for a median of 9.8 weeks (5th percentile 9.0 weeks), indicating this regimen may be effective for a typical prolonged therapy duration of 6–8 weeks. The other alternative regimen of 1,200 mg followed by 800 mg every 14 days resulted in predicted free plasma concentrations above the breakpoint for a median of 7.6 weeks (5th percentile 7.1 weeks) and above the MIC_90_ for a median of 9.1 weeks (5th percentile 8.4 weeks). When compared to the weekly regimens, both every 2-week dosing schemes lead to a total duration of therapy approximately 2 weeks shorter, reducing unnecessary antibiotic exposure after treatment completion. Importantly, this duration is still sufficient for most indications requiring approximately 6 weeks of antibiotic treatment. Because the optimal PK/PD target is not well established for ORI, Table S1 includes other notable PK parameters for each of the simulated dosing regimens.

Both regimens administered every 2 weeks are expected to maintain serum concentrations above the breakpoint throughout the entire dosing interval, indicating that the longer interval still provides therapeutic exposure for the entire duration. Because ORI is often used in patients with difficulty adhering to therapy, it is important that treatment plans have some flexibility for missed appointments or other unexpected issues. To assess this, we modeled a single 1,200 mg dose and evaluated the *f*T > MIC (Fig. S1). After a single dose, 95% of patients should be expected to maintain free serum concentrations above the breakpoint for 19 days and above the MIC_90_ for 30 days. This indicates that, if an every 2-week regimen is planned, there is, conservatively (with the breakpoint as the MIC target), a 5-day cushion to reschedule appointments for the next dose if necessary. Of note, due to accumulation with repeated doses, this cushion may be longer after subsequent doses.

To determine the minimum number of doses required to attain a 6-week duration targeting 100% *f*T > MIC, regimens with fewer total doses were simulated. The predicted free plasma concentrations resulting from these regimens are available in the Supplemental material (Fig. S2). For a dosing strategy of 1,200 mg administered weekly, three doses are required for 95% of patients to achieve a 6-week duration for an isolate at the breakpoint. Two weekly doses of 1,200 mg do not attain this 6-week target. Four doses are required if utilizing a 1,200 mg loading dose, followed by 800 mg administered weekly. For every 2-week regimens, two-dose regimens do not attain a 6-week duration, and three doses are required.

## DISCUSSION

Despite growing use of ORI to manage invasive infections, there is a lack of PK data to support the use of multidose regimens. The most commonly reported regimens utilize weekly dosing, either by repeating weekly 1,200 mg doses or using 1,200 mg as a loading dose followed by 800 mg doses (3, 8, 11). Our data show that utilizing 1,200 mg weekly as a six-dose regimen is expected to result in free plasma concentrations that exceed the breakpoint for >9.6 weeks for the entire confidence interval. Use of lower 800 mg repeat doses led to a reduction of less than 1 week in time of target attainment. This duration far exceeds the intended antibiotic exposure duration at the end of therapy (e.g., 6 weeks) and suggests that these patients may be receiving more ORI than necessary, resulting in higher cost and the potential increased risk of adverse effects.

In an effort to reduce the total amount of drug administered, the ORI dosing regimens were modified to increase the interval to every 14 days (i.e., 1,200 mg every 2 weeks or 1,200 mg as a loading dose followed by 800 mg every 2 weeks), which also reduces the total number of doses to 3. Based on these simulations, the majority of patients are expected to maintain plasma levels above the breakpoint for 7.9 weeks with repeat 1,200 mg doses or 7.1 weeks with 800 mg dosing after a 1,200 mg loading dose. When compared to the weekly dosing regimens, the every-2-week approach reduces the total duration of therapy by ~2 weeks; however, depending on the infection being treated and prior antibiotics administered, this duration is nearly always still adequate for an antibiotic treatment duration of 6 weeks. Furthermore, the modeling suggests that a fourth dose (or more) can also be added onto these every 2-week regimens if longer durations are required, while still reducing the total drug administered.

The use of every 2-week dosing strategies can reduce total drug and administration costs while also increasing convenience for patients by reducing the total number of required appointments. Our study suggests that every 2-week dosing regimens have the potential to achieve these benefits without compromising target attainment. These data can also be used to provide flexibility to patients who may have difficulty adhering to strict weekly dosing schedules. Notably, a single dose is expected to provide therapeutic exposure for the majority of patients for 19 days for an isolate at the breakpoint, providing flexibility for these patients when necessary.

Overall, this study provides many dosing options that can be utilized for patients who often have concerns regarding medication adherence. The decision surrounding which regimen to use should be patient-specific and incorporate the patient’s preferences, availability, reliability, and specific requirements for treatment duration (i.e., continuation regimen after initial therapy). For example, a patient requiring 6 weeks of therapy who has significant difficulties with adherence may be a good candidate for 1,200 mg weekly ×3 doses. This regimen may provide the maximum amount of flexibility, as missed appointments can be rescheduled over a week later, allowing prescribers and patients to make arrangements for a new infusion as necessary. Patients requiring even longer durations (potentially including patients using ORI for long-term suppression) are likely to prefer less frequent, every 2-week appointment options.

Recent infective endocarditis guidelines released by the American Heart Association recommend utilizing ORI at a dose of 1,200 mg on day 1 and day 8 (18). Our simulations indicate that this regimen may fail to achieve a 6-week treatment duration for some patients. This regimen is expected to maintain *f*T > breakpoint for a median of 38 days (5th percentile 35 days) and *f*T > MIC_90_ for a median of 49 days (5th percentile 44 days). Based on these data, a two-dose regimen will likely not achieve a 6-week treatment duration for infections with an MIC at the breakpoint. In these situations (or when the MIC is unknown), we recommend using a third dose or employing an alternative dosing strategy. If a patient receives a week or more of initial antibiotic therapy before initiating ORI, then this two-dose regimen is likely appropriate.

While the breakpoint for *S. aureus* and vancomycin-susceptible *E. faecalis* is 0.12 µg/mL, MICs as high as 0.25 and 0.5 µg/mL have been reported, most often for vancomycin-resistant *Enterococcus faecium* (19). Clinicians may seek further data to guide use of ORI in these cases. Based on the results of these simulations, clinicians should take caution when utilizing multidose ORI for these patients. For all four regimens, 5th percentile trough levels after the first dose fall below 0.25 µg/mL, indicating that some patients will not be expected to be therapeutic for the entire first dosing interval. For the weekly regimens, subsequent trough values remain above 0.25 µg/mL, but not above 0.5 µg/mL. Given that these isolates are often multidrug-resistant and have very limited treatment options, some will still choose to pursue treatment with ORI. There is a published case report of a patient treated with ORI for vancomycin-resistant *E. faecium* with a MIC of 0.5 µg/mL (20). This patient was managed with an initial ORI dosing frequency of every 48 h, a regimen that has also been reported in two other case reports (21, 22). This more frequent dosing may be required for these patients, and therapeutic drug monitoring (TDM) should be considered in these situations if available. For an MIC of 0.25 µg/mL, ORI dosed at 1,200 mg weekly is predicted to provide adequate exposure for the majority of patients, with the lower 5th percentile briefly dropping below the MIC at the end of the first dose. Caution should be taken when utilizing this regimen for patients expected to clear the medication faster (i.e., younger, taller patients based on the model covariates). Again, TDM is recommended if available to ensure serum concentrations remain above the organism MIC.

A limited number of case reports have utilized ORI dosing intervals longer than the traditional 1 week. Redell et al. (10) reported that 14/32 patients treated with multiple doses of ORI were managed using every 2-week dosing schemes, with 13/14 reporting favorable outcomes. All nine patients with chronic osteomyelitis in a study by Chastain et al. (9) were managed with dosing intervals of 14 days or greater and achieved clinical cure. Załęska-Kocięcka et al. (14) reported on seven patients with left ventricular assist device infections treated with multidose ORI with dosing intervals ranging from 14 to 28 days, all with clinical cure. Finally, Vena et al. (12) reported on 20 patients with complicated bloodstream infections or endocarditis managed with multidose ORI. Of these, 10 utilized dosing regimens with every 2-week schedules, and 1 experienced infection recurrence (a patient with prosthetic valve endocarditis who had not undergone surgical intervention). Importantly, *Staphylococcus* spp. were the most common causative organisms across all of the above studies, though *Enterococcus* spp. were also common. Collectively, these data highlight the growing body of evidence supporting the efficacy of multidose ORI regimens with every 2-week dosing intervals.

The primary limitation of this study is the lack of a validated PK/PD target for ORI. While this has been evaluated using a variety of methods, results have been inconsistent. Two neutropenic mouse thigh infection models aimed to establish efficacy and PK/PD targets (23, 24). Boylan et al. (23) determined that *C*_max_ had the strongest correlation to microbiological outcomes, although T > MIC and 24-h area under the curve (AUC)/MIC were also strongly correlated. Okusanya et al. (24) found that AUC_0–72h_ was the strongest predictor of microbiological clearance. Clinical data are similarly conflicting. Bhavnani et al. (25) conducted a study of 55 patients with *S. aureus* bacteremia and found that *f*T > MIC was correlated with both microbiological and clinical success. However, these results are not generalizable due to two main reasons. First, oritavancin was dosed daily based on body weight prior to being developed as a large, single-dose 1,200 mg regimen. Also importantly, the MIC data in this study cannot be extrapolated to current CLSI standards since it did not include the surfactant polysorbate 80 in the media to prevent oritavancin adhesion to polystyrene. Oritavancin MIC90 was reported to be 16- to 32-fold higher when tested in the absence of polysorbate 80 (26). In the literature supporting Food and Drug Administration approval of a single 1,200 mg dose, PK/PD analysis from the SOLO I and II trials identified an AUC_0–72h_/MIC target that was associated with clinical response in patients with skin and soft tissue infections (27). Unfortunately, this correlation was only identified within the first 72 h of therapy, and this target cannot be utilized to determine the optimal timing of subsequent dosing. However, this early target is still relevant for clinicians using ORI as initial therapy. Finally, a single case report that used TDM to optimize ORI dosing intervals utilized a trough target of 3 µg/mL (28). However, it is unclear how this target was obtained, and previous models confirm that it is not attainable for most patients (29).

Because of this conflicting data and the likelihood that *C*_max_, AUC, and T > MIC are all highly correlated given the long half-life of this drug, we opted to utilize 100% *f*T > MIC as the PK/PD target in this study. This approach was also used by Bongiovanni et al. (30) in a case series of two patients receiving ORI for recurrent MRSA osteomyelitis. The managing clinicians opted to use TDM to monitor these patients and target trough values above the organism MIC. Unfortunately, ORI TDM is not currently available in the United States, and therefore, clinicians must rely on population-based data to predict likely drug levels and determine what dosing regimen is best for the patient. In these clinical situations, the acuity and burden of initial infections are lower; therefore, PK/PD goals for these patients may be different than the initial onset. This rationalizes the use of the *f*T > MIC target for prolonged, multidose therapy.

There are three other notable limitations to this study. First, these models predict free plasma concentrations, which do not necessarily correlate with antibiotic concentration at infection sites outside of the bloodstream. An evaluation of ORI bone penetration in rabbits found that drug exposure in bone exceeded that of plasma, with slower clearance noted; though, to our knowledge, this has never been measured in humans (31). Given the significant number of cases reporting the use and success of ORI to treat osteomyelitis, bone penetration is likely to be adequate. Second, the results of this study cannot be extrapolated to patients with ages, heights, and weights outside the ranges included in the original model (7). Finally, we opted to utilize 1-h infusion times (Kimyrsa) across all simulated regimens, as this is the more common administration strategy. However, Kimyrsa is only commercially available as 1,200 mg and cannot be administered as 800 mg over 1 h. In these cases, the 800 mg dosing regimens would need to be given over 3 h as the Orbactiv formulation. While previous PK evaluations have determined no significant change in drug exposure between the two infusion times, caution is warranted when extrapolating these data to the use of a 3-h infusion time (Orbactiv) (16).

In summary, the results of our study support expanding currently utilized dosing intervals for multidose ORI from 7 to 14 days. Both 1,200 mg every 2 weeks for three doses and 1,200 mg as a loading dose followed by 800 mg every 2 weeks for three total doses are expected to maintain free plasma concentrations above the breakpoint for the entire dosing interval. Available case reports of every 2-week ORI use support this strategy and have reported favorable clinical outcomes. Further PK/PD and clinical trial efficacy data are warranted to validate optimal dosing strategies.

## SUPPLEMENTAL MATERIAL

Supplemental material aac.01049-26-s0001.docx Table S1; Fig. S1 and S2.

ASM does not own the copyrights to Supplemental Material that may be linked to, or accessed through, an article. The authors have granted ASM a non-exclusive, world-wide license to publish the Supplemental Material files. Please contact the corresponding author directly for reuse.
