# Supplementary material for: Pharmacokinetic/pharmacodynamic evaluation of multidose oritavancin for prolonged durations of therapy
Source: Antimicrob Agents Chemother. 2026 Oct 14;70(11):e01049-26. doi: 10.1128/aac.01049-26 (PMC13731175; doi:10.1128/aac.01049-26)
Supplement: Supplementary file 1 [file aac.01049-26-s0001.docx]

**Pharmacokinetic/Pharmacodynamic evaluation of multidose oritavancin for prolonged durations of therapy**

Cecilia F. Volk, Max Blumenthal, Nolan Thomas, Wesley D. Kufel, Warren E. Rose, Sin Yin Lim

**Supplemental Materials**

**NONMEM Control File:**

$INPUT C ID TIME AMT RATE DV EVID MDV AGE HTCM

$DATA simulation_data_1200qw.csv IGNORE=C

$SUBROUTINES ADVAN11 TRANS4

$PK

; --- Structural parameters ---

TVV1	= THETA(1) * (AGE/47)**THETA(7)

TVV2	= THETA(2)

TVV3	= THETA(3)

TVCL	= THETA(4) * (HTCM/170)**THETA(8)

TVQ2	= THETA(5)

TVQ3	= THETA(6)

V1 = TVV1 * EXP(ETA(1))

V2 = TVV2 * EXP(ETA(3))

V3 = TVV3 * EXP(ETA(4))

CL = TVCL * EXP(ETA(2))

Q2 = TVQ2 * EXP(ETA(5))

Q3 = TVQ3 * EXP(ETA(6))

S1=V1

$THETA

5.79	;[V1]

75.5	;[V2]

6.29	;[V3]

0.445	;[CL]

0.469	;[Q2]

0.666	;[Q3]

-0.641	;[V_age]

0.695	;[CL_htcm]

$OMEGA BLOCK(6)

0.117873399 ;[P]

0.038070523 ;[F]

0.073774345 ;[P]

0.079243616 ;[F]

0.130062623 ;[F]

0.233712075 ;[P]

-0.071883609 ;[F]

0.119224668 ;[F]

0.188908263 ;[F]

0.389847414 ;[P]

0.004200033 ;[F]

0.124359204 ;[F]

0.207743770 ;[F]

0.292915975 ;[F]

0.256823298 ;[P]

-0.170583197 ;[F]

0.091855734 ;[F]

0.116718824 ;[F]

0.488172795 ;[F]

0.327440444 ;[F]

0.760872549 ;[P]

$SIGMA

0.182	;[P]

0.22	;[A]

$ERROR

IPRE = F

Y = F * (1 + EPS(1)) + EPS(2)

REP = IREP

$SIMULATION (1234567) ONLYSIM SUBPROBLEMS=10

$TABLE REP ID TIME IPRE PRED AGE HTCM

NOPRINT ONEHEADER FILE=simulation_output.tab

**Table S1: Pharmacokinetic parameters associated with each dose of each modeled regimen**

**Figure S1: Simulated oritavancin (ORI) exposure resulting from 1200 mg x1 dose**

Free plasma concentrations after administration of 1200 mg x1 dose. Solid lines indicate the median value and shaded region highlights the 5th-95th percentiles. The breakpoint (0.12 µg/mL) and 90th percentile MIC (MIC90) (0.06 µg/mL) are indicated by red dashed lines.

**Figure S2: Additional ORI Regimen Simulations**

Free plasma concentrations after administration of various oritavancin regimens as labeled above each graph. Solid lines indicate the median value and shaded region highlights the 5th-95th percentiles. The breakpoint (0.12 µg/mL) and 90th percentile MIC (MIC90) (0.06 µg/mL) are indicated by red dashed lines.
